# Insured Fertility Care in Japan: Can Universal Coverage Preserve a Single-Embryo-Transfer Culture?

**DOI:** 10.7759/cureus.114852

**Published:** 2026-08-20

**Authors:** Daichi Inoue

**Affiliations:** 1 Department of Reproductive Medicine, Nishitan-ART Clinic Sakae, Nagoya, JPN

**Keywords:** assisted reproductive technology, health insurance, health policy, in vitro fertilization, japan, multiple pregnancies, single embryo transfer

## Abstract

Japan has one of the world’s lowest fertility rates, and assisted reproductive technology (ART) now accounts for a substantial and growing share of Japanese births. In early 2022, general infertility treatment, ART, and male infertility treatment were incorporated into Japan’s universal health insurance, replacing a means-tested subsidy and largely self-funded model with insured care at a fixed coinsurance rate. The insured pathway is bounded by an upper age limit for initiating treatment, caps on the number of covered embryo transfers that tighten with age, and the prohibition on mixed billing except for designated advanced medical care. This narrative review examines the early clinical implications of the reform for practicing obstetrician-gynecologists by describing national ART outcomes before and after coverage using the Japan Society of Obstetrics and Gynecology (JSOG) registry and placing Japanese practice in an international context. Relevant literature and registry data were identified by searching PubMed and Google Scholar together with the primary Japanese registry and policy sources and the major international ART registries. In the two years after coverage began, registered cycles and ART-derived neonates rose to successive record highs, and access broadened particularly among younger patients, while Japan's characteristic profile of minimal stimulation, freeze-all with frozen-thawed embryo transfer, and predominant single embryo transfer (SET) was largely maintained. However, the overall SET rate declined, and multiple pregnancies increased in the first full insured year, particularly among older patients; these findings are consistent with, but do not prove, a possible incentive effect of fixed transfer caps. Internationally, conventional fresh-cycle live-birth metrics are difficult to interpret for Japan because of its practice pattern and older patient population, whereas its multiple-birth rate remains among the world's lowest. For clinicians, the priorities of the insured era are to raise the age-43 timeline early, defend elective SET near coverage limits, and make explicit the mixed-billing trade-offs of non-covered options such as preimplantation genetic testing (PGT).

## Introduction and background

Japan is confronting one of the most severe demographic contractions in the industrialized world. The total fertility rate fell to a record low of 1.20 in 2023, and annual live births declined to 727,288, placing support for childbearing among the country's foremost priorities [1]. Against this backdrop, assisted reproductive technology (ART) has become integral to Japanese reproductive care: by 2022, roughly one in 10 Japanese newborns was conceived through ART [2]. For obstetrician-gynecologists, infertility care is therefore a substantial and growing component of routine practice.

Japan is also, by volume, one of the world's largest providers of ART. Since the first Japanese in vitro fertilization birth in 1983, activity has grown to more than half a million registered cycles annually, 543,630 cycles yielding 77,206 neonates in 2022, delivered through an unusually dense network of predominantly private clinics and documented in one of the most complete national registries anywhere [2]. Japanese practice is distinctive in style, characterized by minimal ovarian stimulation, a freeze-all approach with frozen-thawed embryo transfer (FET) as the principal route to birth, and a deeply entrenched single embryo transfer (SET) culture producing one of the lowest multiple-birth rates internationally. These features, developed under a self-funded model, form the baseline against which recent policy change must be read.

In April 2022, general infertility treatment, ART, and male infertility treatment were incorporated into Japan's universal health insurance, replacing a means-tested subsidy and largely self-funded model with insured care at the standard 30% coinsurance [3]. This was among the most far-reaching public financing changes for fertility care in any high-income country, but it was not simply a matter of lowering cost. Insured care arrived with a defined list of covered procedures, formal age and cycle-count limits, and the constraints of Japan's prohibition on mixed billing, all of which bear directly on stimulation intensity, embryo-transfer decisions, and the counseling of patients seeking non-covered options.

Two years on, sufficient national data have accumulated for an initial clinical appraisal. This review has two aims: to describe how national ART outcomes evolved before and after coverage and to place Japanese practices in an international context. In plain terms, it asks whether making fertility treatment affordable through public insurance has widened access without eroding the safety gains, above all, Japan's very low rate of multiple pregnancy, that its practice culture had achieved under the previous self-funded model. We outline the reform in clinical terms and the distinctive Japanese practice profile that precedes it, present the before-and-after registry record and the international comparison, consider how funding and regulation shape practice, draw out the clinical implications and controversies, and close with future directions and conclusions. This review is intended as an early clinical interpretation of post-reform national trends and their implications for practice, rather than a formal causal evaluation of the 2022 policy. This narrative review prioritized primary Japan Society of Obstetrics and Gynecology (JSOG) registry reports, official Ministry of Health, Labour and Welfare policy documents, major international registry publications, and selected peer-reviewed studies relevant to embryo transfer policy, insurance-related practice change, and obstetric outcomes after ART.

## Review

Review methods

This article is a narrative review rather than a systematic review, and no formal protocol was registered. To identify relevant material, PubMed and Google Scholar were searched, together with the official publications and websites of the JSOG, the Ministry of Health, Labour and Welfare, and the major international registries (the United States CDC, the United Kingdom Human Fertilisation and Embryology Authority (HFEA), European Society of Human Reproduction and Embryology-European IVF Monitoring Consortium (ESHRE-EIM), Australian and New Zealand Assisted Reproduction Database (ANZARD)). Search terms combined the concepts "assisted reproductive technology," "in vitro fertilization," "insurance coverage" or "reimbursement," "single embryo transfer," "multiple pregnancy," and "Japan," and the reference lists of relevant articles were also examined.

**Figure 1:**
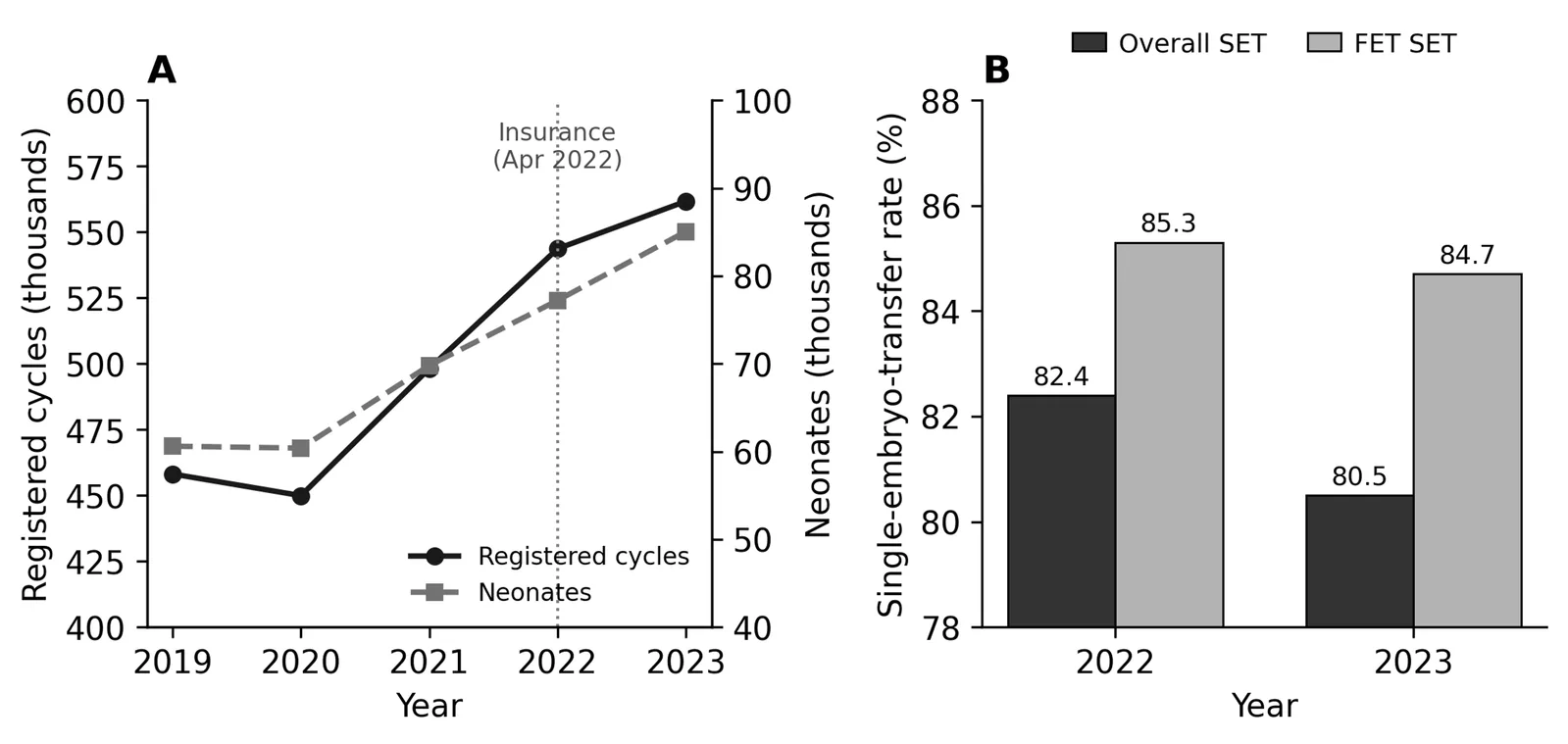
Japanese assisted reproductive technology registry trends across the insurance transition, 2019-2023. (A) Registered treatment cycles (solid line, left axis) and neonates (dashed line, right axis) rose to successive record highs; the dotted vertical line marks the start of public insurance coverage in April 2022. (B) Single-embryo-transfer (SET) rate in 2022 versus 2023: the frozen-thawed embryo transfer (FET) SET rate remained near 85%, whereas the overall (fresh plus frozen) SET rate declined from 82.4% to 80.5% in the first full insured year. Source: Data are from the cited Japan Society of Obstetrics and Gynecology annual ART summary reports [2,6,10-12]. Image credits: Figure 1 is an original figure created by the author using Python (Matplotlib; Python Software Foundation, Beaverton, OR, USA).

For the before-and-after national comparison, priority was given to the primary JSOG annual registry reports, which provide near-complete, cycle-level national data; each year described in the text is sourced to the corresponding JSOG report [2-19]. International comparisons drew on the most recent full report of each national or regional registry. Peer-reviewed studies on embryo-transfer policy, insurance-related practice change, preimplantation genetic testing (PGT), and obstetric outcomes after ART were selected for relevance and methodological quality at the author's discretion, as is appropriate for a narrative synthesis; this selection was not intended to be exhaustive. Consistent with this narrative design, evidence was synthesized descriptively, and no meta-analysis, formal risk-of-bias assessment, or PRISMA-style flow diagram was undertaken because the review integrates heterogeneous sources, national registry reports, government policy documents, and studies with differing designs and outcome definitions, rather than a defined set of comparable studies addressing a single question Figure 1 presents the Japanese ART registry trends across the insurance transition, 2019-2023 from values published in JSOG registry reports [2,6,10-12].

The 2022 reform in clinical terms

Before April 2022, ART in Japan was almost entirely self-funded, a single IVF cycle commonly costing ¥500,000-¥800,000 out of pocket; from 2004 a means-tested subsidy partially offset this, and removal of the subsidy's income ceiling in 2021 expanded uptake in the year preceding the reform [3]. The reform converted this model into insured care at 30% coinsurance across the routine ART sequence (Table 1): general infertility management and intrauterine insemination; oocyte retrieval, in vitro fertilization (IVF), and intracytoplasmic sperm injection (ICSI), culture, cryopreservation, and fresh or frozen-thawed transfer; and surgical sperm retrieval including (micro)TESE [3]. Out-of-pocket cost fell to roughly ¥10,000 per insemination cycle and about ¥200,000 per IVF/ICSI cycle, before the income-based ceiling of the High-Cost Medical Care Benefit [4].

**Table 1:** Insurance coverage tiers for fertility treatments in Japan ART, assisted reproductive technology; ERA, endometrial receptivity analysis; EMMA, endometrial microbiome metagenomic analysis; ALICE, analysis of infectious chronic endometritis; ICSI, intracytoplasmic sperm injection; IMSI, intracytoplasmic morphologically selected sperm injection; IUI, intrauterine insemination; IVF, in vitro fertilization; MHLW, Ministry of Health, Labour and Welfare; PGT-A, preimplantation genetic testing for aneuploidy; PGT-M, preimplantation genetic testing for monogenic disease; PICSI, physiological intracytoplasmic sperm injection; SEET, stimulation of endometrium embryo transfer; TESE, testicular sperm extraction. Combining insured care with non-designated self-pay care renders the entire episode self-pay; only Tier B adjuncts may be combined with an insured cycle. The advanced-care list is revised periodically, and its boundaries move: PGT-A, formerly self-pay in all settings, is now combinable with an insured cycle only at approved facilities and within defined indications (repeated implantation failure, recurrent pregnancy loss or stillbirth, or a confirmed chromosomal structural abnormality in either partner); outside these conditions, its use converts the entire cycle to self-pay. Source: Compiled by the author from Ministry of Health, Labour and Welfare documents on insurance coverage of fertility treatment and the list of advanced medical care technologies [3,5].

No.	Tier	Services	Patient cost
A	Covered by public insurance	General infertility management; intrauterine insemination; oocyte retrieval; IVF and ICSI; fertilization and embryo culture; embryo cryopreservation and storage; fresh or frozen-thawed embryo transfer; surgical sperm retrieval including (micro)TESE	30% coinsurance; income-based monthly ceiling (High-Cost Medical Care Benefit); about 10,000 yen per IUI and about 200,000 yen per IVF/ICSI cycle
B	Advanced medical care (mixed billing permitted)	MHLW-designated adjuncts combinable with an insured cycle, e.g., time-lapse imaging; ERA/ERPeak; EMMA/ALICE; SEET method; two-step transfer; endometrial scratch; PICSI; IMSI; immunoglobulin or tacrolimus for repeated implantation failure; anti-beta-2-glycoprotein-I/neoself antibody testing (indicated for recurrent pregnancy loss); PGT-A, at approved facilities and within defined indications	Insured base at 30% coinsurance plus full self-pay for the advanced component
C	Not covered / self-funded	PGT-M; PGT-A outside approved facilities or defined indications; elective (non-medical) oocyte freezing; third-party reproduction (donor gametes, surrogacy); any treatment started at age 43 years or older or beyond covered transfer limits; renewal of self-pay-frozen embryos	100% self-pay

Insured ART is bounded by rules clinicians must build into counseling (Table 2). Coverage requires the woman to be under 43 at initiation, though a plan established before that birthday may continue; covered embryo transfers are capped by age at initiation-up to six if under 40, three if 40-42-and the count resets after each delivery. There is no male age limit, and both married and common-law couples are eligible [3].

**Table 2:** Eligibility criteria for insured assisted reproductive technology in Japan Source: Compiled by the author from Ministry of Health, Labour and Welfare documents on insurance coverage of fertility treatment [3].

Criterion	Rule
Maternal age at initiation	Under 43 years; a plan established before age 43 years may continue under insurance
Covered transfers, initiation under 40 years	Up to six embryo transfers
Covered transfers, initiation 40-42 years	Up to three embryo transfers
Count reset	Per child; the count resets after a delivery
Partner age	No limit
Relationship status	Legally married and common-law (de facto) couples eligible

The most consequential rule for practice is the general prohibition on mixed billing: combining insured with self-funded care ordinarily renders the entire episode self-pay. The only exceptions are technologies designated as advanced medical care, which may be layered onto an insured cycle, with the patient paying coinsurance for the covered base plus full price for the adjunct; this periodically revised list includes time-lapse imaging, endometrial receptivity and microbiome testing, and specialized sperm-selection techniques (Table 1) [5]. Because non-designated additions forfeit insurance for the whole cycle, several routine conversations become explicit financial trade-offs. PGT for aneuploidy (PGT-A) illustrates how this boundary shifts over time. It is not insured, but following its designation as advanced medical care, it may now be combined with an insured cycle at approved facilities and within defined indications: repeated implantation failure, recurrent pregnancy loss or stillbirth, or a confirmed chromosomal structural abnormality in either partner [5]. Outside those facilities and indications, choosing PGT-A still converts an otherwise insured cycle entirely to self-pay, and embryos frozen under self-pay must be renewed as self-pay thereafter. For the clinician, then, the reform is best understood not as free treatment but as a rule-bound framework that actively shapes clinical decisions.

The Japanese ART practice profile

Japanese ART is delivered through an unusually large, decentralized network of predominantly private clinics; 634 of 635 registered facilities reported to the national registry for 2022, and about 602 performed ART [2]. Practice is documented through the JSOG registry, which has monitored activity since 1986 and collected cycle-specific data since 2007-one of the most complete longitudinal ART datasets anywhere [6].

A defining feature is the prominence of minimal (mild) ovarian stimulation and natural-cycle IVF. Clomiphene-based and natural-cycle protocols, often with a GnRH-agonist trigger, were developed and popularized in high-volume Japanese centers and are used far more widely than in most Western programs, with the explicit aim of reducing ovarian hyperstimulation syndrome and multiple pregnancy [7,8]. A consequence is fewer oocytes and embryos per retrieval, which lowers the apparent output of any single fresh cycle while shifting the path to pregnancy toward accumulated frozen embryos.

Japan has moved to an unusually pronounced freeze-all/FET-dominant pattern. Freeze-all accounted for 57.5% of the 275,296 oocyte retrievals in 2022, and FET is the principal route to birth: the overwhelming majority of ART neonates each year arise from FET rather than fresh transfer (for example, 72,201 of 77,206 in 2022) [2]. In effect, the fresh cycle functions as an embryo-banking step rather than an immediate attempt at pregnancy.

Japan also practices SET very consistently by international standards. Concerned about ART-related multiple births, the Japan Society for Reproductive Medicine issued a guideline in 2007 that capped transfer at three embryos, restricted transfer to a single embryo in first treatment cycles for women under 35, mandated single transfer whenever a good-quality blastocyst was used, and permitted up to two embryos in other cycles under 40 [9]. Uptake has been high and durable, with SET rates near 82%-85% in recent years and a resulting multiple-delivery rate of about 2.6% in 2022 [2,6,10]. Finally, the treated population is older than in many comparators, mean age 37.6 years in 2022, with 38.7% of cycles in women aged 40 or older, and care is predominantly autologous, with third-party reproduction and routine PGT largely outside established practice [2]. Every subsequent analysis must be read against this baseline.

Clinical outcomes before and after coverage

National outcomes are captured by the JSOG cycle-based registry, to which virtually all facilities report; because the annual reports are descriptive, before-and-after comparisons should be interpreted as temporal trends rather than formal causal estimates [2]. Two timeline features are important. First, insurance coverage began in April 2022, so the 2022 data reflect only nine insured months, whereas 2023 represents the first full insured year. Second, removal of the subsidy income ceiling in 2021 had already increased treatment uptake before insurance took effect, meaning that post-reform changes occurred on top of an already rising baseline.

Registered cycles and ART-derived neonates increased across the transition (Table 3; Figure 1A). After a slight COVID-related decline in 2020, cycles rose from 449,900 in 2020 to 498,140 in 2021, and neonates increased from 60,381 to 69,797 [11,6]. In 2022, the first year including insured treatment, cycles reached 543,630 and neonates 77,206 [2]. In 2023, the first full insured year, both measures rose again to record highs, with 561,664 cycles and 85,048 neonates [12]. The more modest increase in cycle volume in 2023, alongside a continued rise in births, is compatible with ongoing conversion of accumulated frozen embryos into pregnancies and live births, although registry data alone do not allow mechanistic inference. Importantly, because these are unadjusted temporal trends, the increases cannot be attributed to the insurance reform itself: they occurred against a baseline already rising after the 2021 removal of the subsidy income ceiling, and may also reflect post-pandemic recovery, shifts in patient case mix, and other secular factors that this descriptive analysis cannot separate.

**Table 3:** Japanese ART registry trends across the insurance transition, 2019-2023 ART, assisted reproductive technology; ET, embryo transfer; FET, frozen-thawed embryo transfer The overall (fresh plus frozen) SET rate was 82.4% in 2022 and 80.5% in 2023. Source: Compiled by the author from the Japan Society of Obstetrics and Gynecology annual ART summary reports [2,6,10-12].

Year	Registered cycles	Neonates	FET cycles	FET neonates	FET single-ET rate
2019 (pre-coverage)	4,58,101	60,598	2,11,597	54,168	85.10%
2020 (pre-coverage, COVID)	4,49,900	60,381	2,15,285	55,503	85.10%
2021 (pre-coverage; subsidy expanded)	4,98,140	69,797	2,39,428	64,679	84.90%
2022 (insurance, partial year)	5,43,630	77,206	2,64,412	72,201	85.30%
2023 (first full insured year)	5,61,664	85,048	2,71,361	80,774	84.70%

The freeze-all/FET pattern that characterizes Japanese ART was maintained after the reform. Freeze-all accounted for 57.5% of oocyte retrievals in 2022, and FET cycles increased further to 271,361 in 2023, generating the large majority of ART-derived neonates in both years [2,12]. Thus, insurance expansion did not appear to shift national practice toward a predominantly fresh-transfer model.

Affordability-related changes in access were also observed. A nationwide facility survey reported a 4.0% overall increase in ART patients after insurance coverage began, with a particularly marked increase among patients aged 25-34 years, while the number of uninsured patients aged 44 years or older declined [13]. These findings are consistent with broader access among younger eligible patients, while also suggesting persistent demand among patients beyond the insurance age limit.

Japan's established safety-oriented profile was largely preserved, but an early warning signal emerged. In 2022, the first and only partially insured year, a dedicated registry analysis found no rise in multiple embryo transfer; the rate fell slightly from 2021 (15.4% to 15.0%), and the multiple-pregnancy rate was essentially unchanged, although the age-specific multiple-embryo-transfer rate rose with maternal age and blastocyst use within multiple transfers increased [14]. A clearer change was observed in 2023, the first full insured year: the FET SET rate remained near 85%, but the overall single-embryo-transfer rate declined from 82.4% to 80.5% (Figure 1B) and the singleton delivery rate fell to 96.5%, indicating an increase in multiple pregnancies, with the rise concentrated among older patients-those with the fewest covered transfers remaining [2,12,14]. Although these descriptive observations do not permit causal attribution, the temporal pattern is compatible with the possibility that fixed transfer caps may create incentives to favour multiple embryo transfer near age or coverage limits, particularly among older patients approaching those limits. These observations describe a temporal association, not a causal relationship. Several alternative explanations could account for the same pattern independently of any incentive effect, including shifts in the age and prognosis mix of treated patients, recovery from pandemic-related disruption of care, facility-level adaptation to the new billing framework, and the continued rise in treatment volume, which itself increases the absolute number of multiple pregnancies. Distinguishing a genuine incentive effect of transfer caps from these confounders would require patient-level, age-stratified, and cycle-specific analyses that the current aggregate registry data do not support, so the proposed link should be regarded as a hypothesis to be tested rather than an established effect. The principal clinical concern is therefore not a collapse of Japan's SET culture, but whether its long-standing safety advantage can be maintained as access broadens under a capped insurance design.

Japanese ART outcomes in the international context

Japan is a high-volume, older-patient system, and its outcomes must be interpreted accordingly (Table 4). The figures in Table 4 are drawn from different reporting years and rest on non-uniform denominators; multiple births are expressed per birth, per delivery, or as twin deliveries reported separately for fresh and frozen transfers, so the table is intended to display this heterogeneity rather than to rank registries against one another, and the values should not be read as directly comparable. In 2022, it registered 543,630 cycles with ART accounting for about one in 10 births, whereas the United States performed 435,426 cycles and the European ESHRE-EIM consortium reported more than one million treatment cycles across 40 countries in 2019 [2,15,16].

**Table 4:** Headline indicators across major ART registries ANZARD, Australian and New Zealand Assisted Reproduction Database; ART, assisted reproductive technology; CDC, Centers for Disease Control and Prevention; ESHRE-EIM, European Society of Human Reproduction and Embryology European IVF Monitoring Consortium; FET, frozen-thawed embryo transfer; HFEA, Human Fertilisation and Embryology Authority; JSOG, Japan Society of Obstetrics and Gynecology; NASS, National ART Surveillance System; SART, Society for Assisted Reproductive Technology; SET, single embryo transfer. (a) Derived from the singleton live-birth rate of 97.4% reported by JSOG for 2022. (b) SET among all embryo-transfer cycles (CDC/NASS definition); SART reports a lower figure using all cycles as the denominator. (c) Most recent year for which a comparable SET figure is published. (d) Approximately one in 16 births in Australia; the corresponding figure for New Zealand is one in 27. Reporting years differ because registries publish with different lags, so each row shows the most recent compiled year; European figures are taken from the most recent full peer-reviewed EIM report (2019). Multiple-outcome denominators are not uniform: Japan and Australia/New Zealand report multiple births per birth, the United States per delivery, and Europe reports twin deliveries separately for fresh and frozen transfers. Direct cross-country comparison is limited by differences in reporting year, case mix, age distribution, embryo-transfer practice, and outcome denominator; raw success percentages should not be interpreted side by side without explicit denominator alignment. Source: Compiled by the author from national and regional ART registry reports [2,15,16,18,19].

Country/registry	Data year	Annual cycles	ART share of births	SET rate	Multiple delivery rate
Japan (JSOG)	2022	5,43,630	About 10%	82.4% (total); 85.3% (FET)	2.6% (a)
USA (CDC/NASS)	2022	4,35,426	2.60%	85.9% (b)	About 4% (per delivery)
Europe (ESHRE-EIM)	2019	1,077,813 (40 countries)	3.00%	55.40%	Twin 11.9% (fresh); 8.9% (FET)
UK (HFEA)	2021	About 76,000	Not reported	About 75% (2019) (c)	5%
Australia/NZ (ANZARD)	2023	1,12,707	About 6.3% (Australia) (d)	94.80%	2.20%

Read naively, Japanese per-cycle success appears low: in comparative analyses spanning 2004-2013, the live-birth rate per fresh cycle was lowest in Japan and highest in the United States [17]. However, this pattern likely reflects practice style and case mix to a substantial extent, rather than allowing a straightforward inference of inferior performance. In Japan, the near-complete shift to freeze-all with deferred FET, the prevalence of minimal stimulation, a strong SET policy, and an older treated population all make live birth per fresh cycle a potentially misleading summary metric. The fresh cycle often functions primarily as an embryo-banking step rather than an immediate attempt at pregnancy, so the denominator itself is imperfectly aligned with the route by which births are actually achieved.

Fairer interpretation therefore requires attention to FET-based and, ideally, cumulative live-birth denominators. In Japan, the majority of ART births arise from FET, and FET outputs have risen year by year [2,12]. Registries that report cumulative outcomes better capture the effectiveness of modern high-SET practice than conventional fresh-cycle metrics alone [18]. Because the United States reports cumulative success per intended retrieval whereas Japan reports largely per-cycle figures, side-by-side percentages are not interchangeable [2,14]. Directly comparable cumulative live-birth estimates are not, however, routinely available for Japan: the JSOG registry has historically reported outcomes per cycle and per transfer rather than per patient across a complete treatment course, so a cumulative figure equivalent to those published by the United States or by Australia and New Zealand cannot at present be extracted for the national dataset. This reporting gap is itself a key limitation of current international comparison and a specific reason why the registry modernization now underway, intended to enable cumulative reporting, will be important.

Japan is among the most successful countries in avoiding multiple pregnancy, though not uniquely so: its multiple-delivery rate of about 2.6% in 2022 is among the lowest reported internationally and is closely matched by the 2.2% recorded in Australia and New Zealand, where single embryo transfer now accounts for nearly 95% of transfers [2,18]. The United Kingdom reduced its ART multiple-birth rate to 5% in 2021 after a sustained "one at a time" campaign [19]. Continental Europe remained higher, although continuing to decline with broader uptake of SET [15]. Cross-registry comparison remains difficult because registries differ in reporting year, denominator (per initiated cycle, per retrieval, per transfer, or cumulative), outcome definition, and completeness, and conventional per-cycle reporting may systematically understate a patient's true cumulative chance of live birth [18,20]. Raw percentages should therefore not be placed side by side without stating each denominator explicitly (Table 5).

**Table 5:** Live-birth-rate denominators used by major ART registries ANZARD, Australian and New Zealand Assisted Reproduction Database; ART, assisted reproductive technology; CDC, Centers for Disease Control and Prevention; ESHRE-EIM, European Society of Human Reproduction and Embryology European IVF Monitoring Consortium; FET, frozen-thawed embryo transfer; HFEA, Human Fertilisation and Embryology Authority; JSOG, Japan Society of Obstetrics and Gynecology; NASS, National ART Surveillance System. Because registries use different denominators and cumulative conventions, headline live-birth or success percentages are not directly comparable across registries. Source: Compiled by the author from national and regional ART registry reports [2,15,16,18,19].

Registry	Typical denominator	Cumulative
Japan (JSOG)	Per cycle or per transfer, fresh versus FET	No
USA (CDC/NASS)	Per intended or actual retrieval, per transfer	Yes
Europe (ESHRE-EIM)	Delivery or pregnancy per aspiration and per transfer	No
UK (HFEA)	Per embryo transferred or per cycle	Partly
Australia/NZ (ANZARD)	Live birth per initiated or stimulated cycle	Yes

How funding and regulation shape practice

International experience suggests that how ART is financed influences not only access but also clinical practice, including embryo-transfer decisions and multiple-pregnancy risk [21,22]. Two policy levers are especially important. The first is the generosity of funding, including the extent to which treatment costs and repeated cycles are publicly supported. Comparative work indicates that lower out-of-pocket costs generally increase utilization, but that funding expansion alone does not guarantee favorable safety outcomes [21]. The second lever is embryo-number policy, whether imposed through regulation or reinforced through professional norms such as elective SET. Systems that combine broad access with strong SET-oriented practice have been more successful in expanding treatment while limiting multiple births [19,21].

Evidence from other countries illustrates this distinction. In the United States, more generous mandated insurance coverage has been associated with greater treatment use and, in some analyses, with increases in multiple births despite fewer embryos being transferred per cycle on average, likely because expanded access also increases treatment among older and poorer-prognosis patients [22]. In the United Kingdom, by contrast, the "one at a time" campaign increased SET uptake substantially and reduced multiple births without an apparent reduction in overall birth rates [19]. These experiences suggest that funding and embryo-transfer policy should not be treated as interchangeable: the first affects who enters treatment, whereas the second helps determine whether expanded access is achieved with or without a rise in treatment-related multiple pregnancy.

This distinction is particularly relevant to Japan's post-2022 system. The reform substantially reduced financial barriers for eligible patients, but it also introduced fixed caps on covered embryo transfers. In principle, such caps may create pressure to maximize the chance of success within a limited number of insured opportunities, especially among older patients or those approaching eligibility limits. Within this framework, the post-reform decline in overall SET and the increase in multiple pregnancies observed in 2023 can be interpreted as an early signal that capped coverage may work against SET incentives in some patient groups [12,14]. However, current national data remain descriptive, and longer follow-up with more granular age-stratified and cycle-specific analyses will be needed before concluding that the insurance design itself altered transfer behavior. For Japan, the key policy challenge is therefore not simply sustaining funding, but ensuring that the country's historically strong SET culture remains an active counterweight to any incentive created by capped coverage.

Clinical implications and controversies

The 2022 insurance reform translates into several practical tasks for clinicians, each accompanied by some degree of controversy.

Eligibility and the Age-43 Limit

The age-43 initiation limit creates a hard eligibility threshold. Its rationale is grounded in prognosis and resource stewardship, as success rates decline and miscarriage rates increase with advancing maternal age; however, critics argue that a rigid age cutoff may overlook individual variation in ovarian reserve, embryo availability, and prior treatment history [2,4]. In practice, this means that clinicians should raise the insurance timeline early in consultation, so that eligible patients can establish a treatment plan before reaching the age limit.

Transfer Caps and SET

The introduction of fixed caps on covered embryo transfers may create tension with Japan's long-standing SET culture. As discussed above, early post-reform registry trends suggest a decline in overall SET and an increase in multiple pregnancies, particularly among older patients with fewer covered transfers remaining [12,14]. Although these data do not establish causality, they raise a clinically important concern: when insured opportunities are limited, both patients and clinicians may feel pressure to maximize the chance of pregnancy within each remaining cycle. The following practical suggestions reflect the author's clinical perspective rather than conclusions derived directly from the observational data. From this standpoint, Japan's low multiple-pregnancy rate is an achievement worth protecting deliberately, which in practice argues for maintaining elective SET as the default approach wherever feasible, even when patients approaching age or coverage limits request transfer of more than one embryo, and ensuring that any deviation from SET is based on clearly documented shared decision-making.

Mixed Billing and PGT

Because mixed billing is generally prohibited, choosing a non-designated intervention forfeits insurance coverage for the entire cycle, turning several routine discussions into explicit financial trade-offs. PGT is the clearest example. The clinical debate over PGT-A remains unsettled, and the balance of benefit and harm appears to depend heavily on the patient population. In women of advanced maternal age, or those with recurrent pregnancy loss or repeated implantation failure, selected studies suggest it may reduce miscarriage and shorten the time to a live birth by prioritizing euploid embryos for transfer [23,24]. In good-prognosis and unselected populations, by contrast, the largest contemporary randomized trials and cumulative-outcome analyses have not demonstrated a consistent improvement in cumulative live birth, and have raised concern that potentially viable embryos may be discarded, particularly where few embryos are available, as is common under Japan's minimal-stimulation practice [25-28]. On current evidence, any clinically meaningful benefit is therefore most plausible in older or poor-prognosis patients with sufficient embryos to test, whereas routine application to younger, good-prognosis patients is not supported. In Japan, this scientific debate is compounded by a financial one whose terms have recently changed: since PGT-A was designated as advanced medical care, it may be combined with an insured cycle at approved facilities and within defined indications, whereas outside those conditions it still converts an otherwise insured cycle to full self-pay. Clinicians therefore need to establish whether their own facility and the individual patient meet those conditions before framing the trade-off. Similar counseling challenges arise in relation to fertility preservation and third-party reproduction, both of which remain outside routine insurance coverage.

Obstetric Implications of FET

Beyond these financial and procedural questions, clinicians should also recognize that the safety of the post-reform ART landscape cannot be judged solely by whether multiple pregnancy remains rare. As insured access expands within a system already centered on FET, the obstetric implications of a growing FET-conceived population become increasingly relevant to general obstetrician-gynaecologists. Although widespread use of elective SET and FET has substantially reduced twin gestations, observational studies have associated FET pregnancies with a different obstetric risk profile from fresh-transfer pregnancies, including higher rates of hypertensive disorders of pregnancy, large-for-gestational-age infants, and some placental complications [29-33]. These are associations rather than established causal effects, and they are not uniform across FET protocols: the excess risk of hypertensive disorders in particular has been linked specifically to programmed (hormone-replacement) cycles, in which the absence of a corpus luteum is thought to play a role, whereas natural or modified-natural cycles appear to carry a lower risk. This distinction is clinically relevant, since it suggests that protocol choice, rather than FET itself, may account for part of the excess risk. The post-reform expansion of ART therefore has implications beyond infertility treatment itself: as more pregnancies are achieved through insured ART, antenatal surveillance and delivery planning may increasingly need to take account of the distinctive maternal and perinatal features of FET-conceived singleton pregnancies. Preserving Japan's safety advantage in the insured era will require attention not only to embryo number at transfer but also to the broader obstetric consequences of an increasingly FET-based treatment landscape.

Coverage standardization and remaining gaps

The defined list of covered procedures standardizes treatment across a large number of predominantly private clinics. Supporters may view this as establishing a national quality floor, whereas critics may see it as constraining individualized management in complex or poor-prognosis cases. Finally, clinicians will continue to encounter patients whom the current scheme does not adequately reach, including those initiating treatment at age 43 or older, those who have exhausted covered transfers, same-sex couples and single women, and those seeking donor gametes or surrogacy. Candid counseling about these boundaries and gaps is part of clinical practice under the new insured framework.

Limitations and future directions

This review has several limitations. It is a narrative rather than a systematic synthesis, and the national before-and-after comparison rests on aggregate registry data that are descriptive and unadjusted; the observed post-reform changes therefore represent temporal associations that cannot be attributed to the insurance reform itself, given concurrent influences such as the 2021 subsidy expansion, post-pandemic recovery, and shifts in patient case mix. Directly comparable cumulative live-birth estimates are not currently available for Japan, and cross-registry comparisons are further constrained by differences in reporting year, denominator, and outcome definition. Several of the clinical implications drawn here reflect the author's interpretation and clinical perspective rather than conclusions derived directly from the data. These limitations should be borne in mind when interpreting the trends described.

The 2022 reform is an iterative process, not an endpoint. Coverage is revised through biennial fee-schedule updates, and incremental changes have followed: anti-Müllerian hormone testing, insured from 2022 for planning ovarian stimulation, [3] had its indication broadened in 2024 to the assessment of ovarian function, and a sperm-cryopreservation management fee was newly introduced in the same revision, [34] while the advanced-care list continues to change [5]. Some municipalities have begun layering local subsidies onto insured care, covering both the residual 30% coinsurance and the full cost of designated advanced medical care, so that effective patient cost and practical access to adjuncts now vary by place of residence [35]. Clinicians should expect the coverage boundary and effective patient cost to keep shifting.

Robust evaluation depends on the registry, whose modernization is a stated priority for around 2026 [12]. Two enhancements matter most: routine cumulative live-birth reporting, to allow fair international comparison and more accurate counseling; and linkage of ART records to obstetric and neonatal outcomes, to monitor the safety consequences of any change in transfer practice. Key questions remain open-the causal effect of transfer caps on embryo-number decisions, long-term maternal and neonatal outcomes of the post-reform cohort, the role of PGT-A within Japanese practice, and the equity consequences for excluded patients. For the practising clinician, four indicators will signal how the reform matures: the overall SET and multiple-birth rates, the age structure of treated patients, each biennial revision to covered services and limits, and the availability of local subsidies.

## Conclusions

Japan's incorporation of ART into universal health insurance is best understood not as a simple expansion of access but as a natural experiment in whether a health system can broaden fertility care while preserving the safety gains, above all a very low rate of multiple pregnancy, that its practice culture had achieved under a self-funded model. The central lesson for policy is that the generosity of funding and the rules governing embryo number are distinct levers that must move together: lowering financial barriers widens who can be treated, but it does not, on its own, protect against the multiple pregnancies that can follow when limits on covered treatment create pressure to transfer more than one embryo. Where capped public coverage and a strong SET culture coexist, that culture must be actively defended rather than assumed to be self-sustaining.

For the practising obstetrician-gynecologist, this reframes the reform as a set of continuing clinical responsibilities rather than a finished policy: raising the question of eligibility and its time limits early in care, treating elective SET as a default to be protected rather than a norm to be relaxed under pressure, and making the trade-offs of non-covered options explicit so that patients can decide with full understanding. Whether a capped but publicly funded system can sustain both wide access and a high standard of safety is a question of international relevance, and Japan's continuing experience offers a template from which other countries extending public support for fertility care can learn.

## References

[1] (2026). Vital statistics of Japan 2023 (Article in Japanese). https://www.mhlw.go.jp/toukei/saikin/hw/jinkou/kakutei23/dl/03_h1.pdf.

[2] Katagiri Y, Jwa SC, Kuwahara A (2024). Assisted reproductive technology in Japan: a summary report for 2022 by the Ethics Committee of the Japan Society of Obstetrics and Gynecology. Reprod Med Biol.

[3] (2026). Insurance coverage of fertility treatment in Japan (Article in Japanese). https://www.mhlw.go.jp/content/10808000/000929827.pdf.

[4] Okawara M, Kuwazuru T, Masunaga M, Fujimoto K, Nagata M, Iwasa T, Fujino Y (2026). Utilization of fertility treatment in Japan during the first year of insurance coverage: analysis of real-world health claims data. Reprod Med Biol.

[5] (2026). Overview of advanced medical care technologies (Article in Japanese). May.

[6] Katagiri Y, Jwa SC, Kuwahara A (2024). Assisted reproductive technology in Japan: a summary report for 2021 by the Ethics Committee of the Japan Society of Obstetrics and Gynecology. Reprod Med Biol.

[7] Kato K, Takehara Y, Segawa T (2012). Minimal ovarian stimulation combined with elective single embryo transfer policy: age-specific results of a large, single-centre, Japanese cohort. Reprod Biol Endocrinol.

[8] Teramoto S, Kato O (2007). Minimal ovarian stimulation with clomiphene citrate: a large-scale retrospective study. Reprod Biomed Online.

[9] Ethics Committee, Japan Society for Reproductive Medicine (2026). Ethics committee report: guidelines for the number of embryos to be transferred to prevent multiple pregnancies. Tokyo: JSRM; 16 March.

[10] Katagiri Y, Jwa SC, Kuwahara A (2022). Assisted reproductive technology in Japan: a summary report for 2019 by the Ethics Committee of the Japan Society of Obstetrics and Gynecology. Reprod Med Biol.

[11] Katagiri Y, Jwa SC, Kuwahara A (2023). Assisted reproductive technology in Japan: a summary report for 2020 by the Ethics Committee of the Japan Society of Obstetrics and Gynecology. Reprod Med Biol.

[12] Katagiri Y, Jwa SC, Kuwahara A (2026). Assisted reproductive technology in Japan: a summary report for 2023 by the Committee on Professional Scientific Conduct and Clinical Ethics of the Japan Society of Obstetrics and Gynecology. Reprod Med Biol.

[13] Wada A, Yamada M, Shirasawa H, Jwa SC, Kuroda K, Harada M, Osuga Y (2025). Impact of insurance coverage on access to assisted reproductive technology: a nationwide survey in Japan (the IZANAMI project). J Obstet Gynaecol Res.

[14] Ito A, Jwa SC, Kuwahara A (2025). Impact of health insurance coverage for assisted reproductive technology on multiple embryo transfers and multiple pregnancies in Japan. J Obstet Gynaecol Res.

[15] Smeenk J, Wyns C, De Geyter C (2023). ART in Europe, 2019: results generated from European registries by ESHRE†. Hum Reprod.

[16] Kushnir VA, Barad DH, Albertini DF, Darmon SK, Gleicher N (2017). Systematic review of worldwide trends in assisted reproductive technology 2004-2013. Reprod Biol Endocrinol.

[17] (2026). ART national summary figures. https://www.cdc.gov/art/php/national-summary/index.html.

[18] (2026). Fertility treatment 2021: preliminary trends and figures. Fertility treatment.

[19] Kotevski DP, Newman JE, Chaitarvornkit A (2026). Assisted reproductive technology in Australia and New Zealand 2023. Assisted reproductive technology in Australia and New Zealand.

[20] Griesinger G, Larsson P (2023). Conventional outcome reporting per IVF cycle/embryo transfer may systematically underestimate chances of success for women undergoing ART: relevant biases in registries, epidemiological studies, and guidelines. Hum Reprod Open.

[21] Chambers GM, Sullivan EA, Ishihara O, Chapman MG, Adamson GD (2009). The economic impact of assisted reproductive technology: a review of selected developed countries. Fertil Steril.

[22] Zaresani A, Schmidt L (2024). Unintended consequences of policy interventions: evidence from mandated health insurance coverage for IVF treatment. Am J Health Econ.

[23] Forman EJ, Hong KH, Ferry KM (2013). In vitro fertilization with single euploid blastocyst transfer: a randomized controlled trial. Fertil Steril.

[24] Rubio C, Bellver J, Rodrigo L (2017). In vitro fertilization with preimplantation genetic diagnosis for aneuploidies in advanced maternal age: a randomized, controlled study. Fertil Steril.

[25] Munné S, Kaplan B, Frattarelli JL (2019). Preimplantation genetic testing for aneuploidy versus morphology as selection criteria for single frozen-thawed embryo transfer in good-prognosis patients: a multicenter randomized clinical trial. Fertil Steril.

[26] Yan J, Qin Y, Zhao H (2021). Live birth with or without preimplantation genetic testing for aneuploidy. N Engl J Med.

[27] Kucherov A, Fazzari M, Lieman H, Ball GD, Doody K, Jindal S (2023). PGT-A is associated with reduced cumulative live birth rate in first reported IVF stimulation cycles age ≤ 40: an analysis of 133,494 autologous cycles reported to SART CORS. J Assist Reprod Genet.

[28] Cornelisse S, Zagers M, Kostova E, Fleischer K, van Wely M, Mastenbroek S (2020). Preimplantation genetic testing for aneuploidies (abnormal number of chromosomes) in in vitro fertilisation. Cochrane Database Syst Rev.

[29] Chih HJ, Elias FT, Gaudet L, Velez MP (2021). Assisted reproductive technology and hypertensive disorders of pregnancy: systematic review and meta-analyses. BMC Pregnancy Childbirth.

[30] Maheshwari A, Pandey S, Amalraj Raja E, Shetty A, Hamilton M, Bhattacharya S (2018). Is frozen embryo transfer better for mothers and babies? Can cumulative meta-analysis provide a definitive answer?. Hum Reprod Update.

[31] Busnelli A, Schirripa I, Fedele F, Bulfoni A, Levi-Setti PE (2022). Obstetric and perinatal outcomes following programmed compared to natural frozen-thawed embryo transfer cycles: a systematic review and meta-analysis. Hum Reprod.

[32] Matsuzaki S, Nagase Y, Takiuchi T (2021). Antenatal diagnosis of placenta accreta spectrum after in vitro fertilization-embryo transfer: a systematic review and meta-analysis. Sci Rep.

[33] Zaat TR, Kostova EB, Korsen P, Showell MG, Mol F, van Wely M (2023). Obstetric and neonatal outcomes after natural versus artificial cycle frozen embryo transfer and the role of luteal phase support: a systematic review and meta-analysis. Hum Reprod Update.

[34] (2026). Outline of the FY2024 revision of the medical fee schedule: medical technologies (Article in Japanese). Tokyo: MHLW; 5 March.

[35] (2026). Overview of the Tokyo Metropolitan Government’s infertility treatment cost subsidy program (Article in Japanese). https://www.fukushi.metro.tokyo.lg.jp/kodomo/kosodate/josei/funin-senshiniryou/gaiyou.

